# Long‐Term Maintenance of High Neutralizing Anti‐SARS‐CoV‐2 Antibodies Titres in Mares’ Milk and Offspring Serum After Pregnant Mares Immunization With SARS‐CoV‐2 Spike Protein

**DOI:** 10.1002/vms3.70488

**Published:** 2025-07-23

**Authors:** Victor Augusto Roncaglia‐Pereira, Carlos Henrique Dumard, Marcos Monteiro‐Machado, Paulo A Melo, Juliana Fonseca, Leonardo Meirelles, Luis Cunha‐Ribeiro, Patrícia Souza, Jerson Lima da Silva, Leda Castilho, Andréa Cheble de Oliveira, Andre Marco de Oliveira Gomes, Marcelo Abrahão Strauch

**Affiliations:** ^1^ Institute of Medical Biochemistry Leopoldo de Meis and National Institute of Science and Technology for Structural Biology and Bioimaging Federal University of Rio de Janeiro (UFRJ) Rio de Janeiro Rio de Janeiro Brazil; ^2^ Laboratório de Farmacologia das Toxinas Institute of Biomedical Sciences, Federal University of Rio de Janeiro Rio de Janeiro Brazil; ^3^ Vital Brazil Institute Niterói Rio de Janeiro Brazil; ^4^ Cell Culture Engineering Laboratory, COPPE Federal University of Rio de Janeiro (UFRJ) Rio de Janeiro Rio de Janeiro Brazil; ^5^ Institute of Biological and Health Sciences, Department of Physiological Sciences Federal Rural University of Rio de Janeiro Seropedica Brazil

**Keywords:** foals, immunoglobulin G, mares, passive immunization, SARS‐CoV‐2

## Abstract

In this study, we aim to report the persistent presence of anti‐SARS‐CoV‐2 immunoglobulins in pre‐immunized mare offspring. Three mares from Vital Brazil Institute were pre‐immunized with recombinant SARS‐CoV‐2 spike protein and became pregnant during this period. After parturition, the mares’ serum and colostrum/milk and foal serum were collected over 6 weeks. Our results have shown high and persistent presence of IgG and neutralizing antibodies over the weeks not only in the mares’ serum, as expected, but in mares’ colostrum/milk and foal serum as well—what were very surprising. This peculiar approach, which we were able to track specifically antibodies produced from an antigen inoculated only in the pregnant mares’, is distinct from the usual methodology applied in the current reports in this field. Thus, together, these data and our methodology could lead to new approaches to better understanding about equine passive immunization by newborn's breast‐feeding.

## Graphical abstract

This study investigated the total and neutralizing antibodies transferred to offspring from three mares from Vital Brazil Institute that were pre‐immunized with recombinant SARS‐CoV‐2 trimeric spike protein. They became pregnant during the immunization protocol. After parturition, the mare's serum and colostrum and foal serum showed high total and neutralizing antibodies against SARS‐CoV‐2 over 6 weeks.

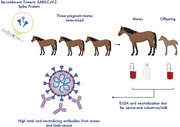

## 1 Introduction

Due to the mares’ epitheliochorial placenta, which do not allow the difusion of large molecules (in which are included the immunoglobulins), foals are born immunosuppressed, therefore, it is essential to transfer antibodies from the mother to the offspring through the colostrum, preventing the emergence of diseases in early life (Erhard et al. 2001; McGuire et al. 1977; Paradis 2006; Perkins and Wagner 2015). It has been described in the literature that immunoglobulin transfer occurs critically during the first 24 h of life, and after this period, antibody levels drastically reduce in milk (Jeffcott 1974b), and the ingested IgG can protect the offspring along 2 months (Kohn et al. 1989; Morris et al. 1985). In addition, antibodies transferred by colostrum can remain present in foals until the first 5 months of life and the endogenous antibodies increases are inversely proportional as the passive antibodies decrease over the 5 months (Baptista et al. 2020; Erhard et al. 2001; Jeffcott 1974a, 1974b).

Although the literature is vast in studies on the passive immunization of horse foals, there is a scarcity of long‐term studies with pregnant mares either during or post a period of immunization with a specific antigen, and this particular context is of crucial importance due to the high usage of horses and mares for hyperimmune serum production, once it has been used for over 100 years as a treatment against accidents with venomous animals and also against infectious diseases (Graham and Ambrosino 2015; Guidolin et al. 1998; Squaiella‐Baptistão et al. 2018).

In a previously published article, our group developed an anti‐SARS‐CoV‐2 biological input produced from immunoglobulin fragments F(ab′)_2_ from horses immunized with the heterologous spike protein trimer of SARS‐CoV‐2. Our immunization strategy consisted of using male and female animals, with three unexpectedly becoming pregnant during the period in which the animals were receiving the antigen. However, as described above, in the literature, it is described that the foals are agammaglobulinemic at birth and the transfer of immunoglobulins from progenitress colostrum is essential during the neonatal period, and although there are many reports in literature on equine passive immunization, these articles are based on total IgG quantification, and our approach could indicate antibodies specifically generated from an antigen inoculated only in pregnant mares. Thus, in this case report, we characterize the presence of SARS‐CoV‐2 immunoglobulins G and neutralizing immunoglobulin G in the offspring of recombinant SARS‐CoV‐2 spike protein‐immunized mares during 6 weeks after birth.

## 2 Methods

### 2.1 Animal Immunization and Blood, Colostrum and Milk Collection

Three 3‐ to 5‐year‐old healthy mares from the IVB farm (Instituto Vital Brazil, Cachoeira de Macacu, Niterói, RJ, Brasil), weighing approximately 350 kg each, were subcutaneously prime‐immunized six times at 7‐day intervals (on Days 0, 7, 14, 21, 28 and 35) and re‐immunized every 2 months for 1 year in different positions of the dorsal region. The animals were subjected to two complementary immunization protocols, described here as C1 and C2. Protocol C1 consisted of the administration of 5 doses of the SARS‐CoV‐2 trimeric spike protein every 7 days, while protocol C2 consisted of the administration of 3 doses. Each immunization of each horse consisted of 200 µg of recombinant SARS‐CoV‐2 trimeric S protein mixed with Montanide ISA 50V adjuvant (Seppic, France) to form an emulsion (one part immunogen in sterile saline to one‐part sterile adjuvant). Recombinant SARS‐CoV‐2 trimeric spike protein expression and purification were described previously in Alvim et al. (2020) and Cunha et al. (2021). The immunization cycles were administered 2 months apart after the start of the first cycle. The individual information on the immunization program for each animal before and during pregnancy is described in Table 1. The mares entered the breeding season and gave birth to three donkeys. Blood from foals and mares, in addition to colostrum and milk from mares, were collected on the day of parturition and at 6 weeks postpartum. Blood collection was performed by venipuncture of the jugular vein, and colostrum and milk were collected by milking the mares. Always prioritizing animal welfare and because these are lactating animals, the veterinary team decided not to immunize and collect blood from mare 834 for five weeks (out of a total of six ‐ a small blood sample was collected in week 3). It was possible to collect a small blood sample from animal 834, which allowed us to perform the microneutralization assay. Although completely healthy, these animals were more hyper‐reactive to the procedures.

**TABLE 1:** Individual details of mare immunization cycles.

Animal	834	835	839
Cycles pre‐pregnancy (start date)		C1 (27/05/2020)	C1 (08/07/2020)
Cycles during pregnancy	C1 (08/07/2020) C2 (30/09/2020) C2 (25/11/2020) C2 (20/01/2021)	C2 (26/08/2020) C2 (21/10/2020) C2 (16/12/2020) C2 (24/02/2021) C2 (26/05/2021)	C2 (30/09/2020) C2 (25/11/2020) C2 (20/01/2021) C2 (26/05/2021)
Date of parturition	01/05/2021	22/06/2021	29/06/2021

Mare immunization cycles: C1 (Initial immunization protocol): 5 doses of 200 µg of SARS‐CoV‐2 trimeric spike protein every 7 days; C2 (Booster immunization protocol): 3 doses of 200 µg of SARS‐CoV‐2 trimeric spike protein every 7 days. In parentheses: Start date of each cycle (dd/mm/yyyy).

### 2.2 Enzyme‐Linked Immunosorbent Assay (ELISA)

In brief, polystyrene high‐adsorption 96‐well microplates (ThermoFisher, USA) were coated with 500 ng/well recombinant SARS‐CoV‐2 S protein (100 µL/well at 5 µg/mL) in carbonate‐bicarbonate buffer (pH 9.6) overnight at room temperature and then blocked with 3% BSA (Sigma, USA) in PBS for 2 h at 37°C. Serially twofold diluted serum samples from three mares and their offspring (from 1:1000 to 1:1.000.000) and the milk of the three mares collected from the first day after giving birth to the fifth week of birth were added to the plate and incubated at 37°C for 1 h. Horseradish peroxidase‐conjugated rabbit anti‐horse IgG (Sigma A6917, EUA) diluted 1:10.000 in PBS was incubated at 37°C for 1 h. After each step, the plates were washed three times with PBST (PBS containing 0.05% Tween 20). Substrate OPD (100 µL/well—Sigma, EUA) was added to the wells and incubated in dark for 10 min in room temperature. The reaction was stopped with the addition of 50 µL of 30% H_2_SO_4_ (v/v) per well, and the absorbance value was measured at *λ* 490 nm in a microplate reader (Epoch/2 microplate Teaser da Biotek). IgG antibody titre was defined as the highest dilution of serum when the *λ* 490 ratio was greater than 2.0, in the same dilution (*λ* 490 of sample each horse/*λ* 490 of negative control). The analyses were carried out in duplicate and data analyses were performed with GraphPad Prism 9.0.

### 2.3 Cell and Virus

African green monkey kidney (Vero, subtype E6) cells were cultured at 37°C in high‐glucose DMEM with 10% foetal bovine serum (HyClone/Cytiva, USA), 100 U/mL penicillin and 100 µg/mL streptomycin (Thermo Fisher, USA) in a humidified atmosphere with 5% CO_2_.

SARS‐CoV‐2 was prepared in Vero E6 cells. All procedures related to virus culture were handled in a biosafety level 3 (BSL3) multiuser facility according to WHO guidelines. Virus titres were determined as plaque‐forming units (PFU)/mL. The virus strain was sequenced to confirm identity, and the complete genome is available in GenBank (SARS‐CoV‐2/human/BRA/RJ01/2020, #MT710714). The Gamma strain (P.1 in pangolin classification) was donated by the Laboratory of Medical Investigation in Virology from University of Sao Paulo (EPI_ISL_1060902–GISAID). The clarified virus stocks were kept at −80°C.

### 2.4 Microneutralization Assay

To assess the neutralization titre, the samples (previously inactivated by warming at 56°C for 30 min) with twofold consecutive serial dilutions were incubated with 100 PFU of prototype SARS‐CoV‐2 or SARS‐CoV‐2 Gamma variant for 1 h at 37°C. Then, the samples were transferred to 96‐well plates with monolayers of Vero cells (2 × 10^4^ cells/well) for 1 h at 37°C. Cells were washed, and fresh medium with 2% FBS and 2.4% CMC was added. On Day 3 postinfection, the cytopathic effect was scored in at least 2 replicates per dilution by independent readers. The readers were blind with respect to the sample ID.

## 3 Results

### 3.1 Transfer of Colostral Passive Immunity From IgG Anti‐Sars‐CoV‐2

**FIGURE 1:**
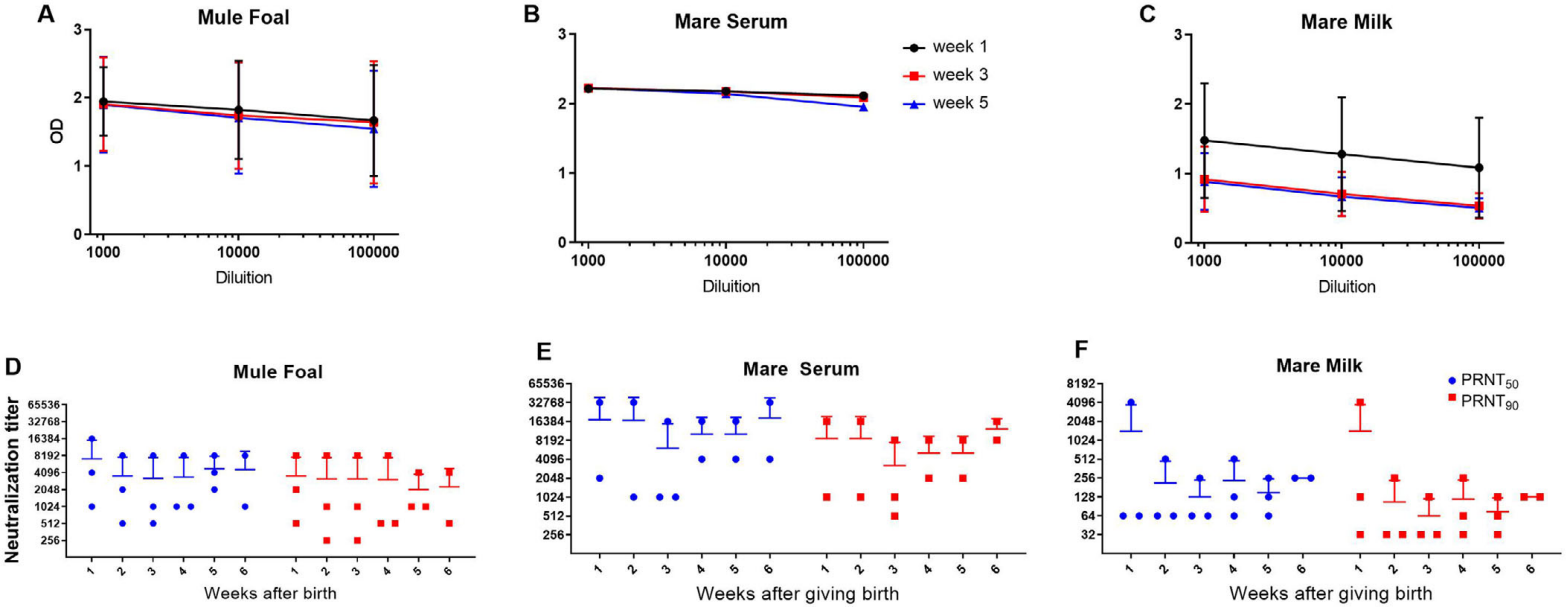
Anti‐S antibodies measured by ELISA and microneutralization assays in different samples collected over time. Titration of foals serum (A), mare milk (B) and mare serum antibodies (C). Each symbol represents one animal (834‐circle/835‐square/839‐triangle) and each colour represents a week (Week 1‐black/Week 3‐red/Week 5‐blue). MicroPRNT_50_ and microPRNT_90_ for foals (D) and mares’ serum (E) and milk (F) collected on Weeks 1–6 after farrowing.

During the process of immunization of horses, three mares were mated with a donkey and fertilized, which continued to receive the antigen as described in the methodology, all mares after 11 months in the immunization proce, the last immunization dose of mare 835 being administered 2 months before the foaling and mares 834 and 839 received the last dose 3 months before the foals were born. All mares subjected to the immunization process for 6‐12 months (as described in Table 1) presented high titers of IgG Anti‐SARS‐CoV‐2 in both colostrum and milk. Using ELISA, we demonstrated effective colostral transfer of anti‐SARS‐CoV‐2 IgG to the newborn donkeys, which subsequently presented antibody titers comparable to those found in the serum and milk of the immunized mares (Figure 1A–C). Although the complete quantification of serum Anti‐Sars‐CoV‐2 IgG was only possible for mare 835 ‐ as described in the methodology ‐ a previous study published by our group (Cunha et al., 2021) showed that immunization of horses and mares with the heterologous Sars‐CoV‐2 trimeric spike protein undoubtedly induces a large production of specific antibodies in these animals. Moreover, the high concentrations of total and neutralizing antibodies detected in the milk of immunized mares provide strong evidence about the antibody production capacity of these animals against the administered antigen.

### 3.2 Immunoglobulin Transfer Through Colostrum Generates High Neutralizing Titres in the Offspring Serum of Immunized Mares

We assessed the in vitro neutralization of serum and milk from mares immunized with heterologous SARS‐CoV‐2 Spike protein and its offspring for 6 weeks from foal birth against the ancestral strain of SARS‐CoV‐2. First, we performed the microneutralization assay with foal serum. The results pointed to high neutralizing titres during the 6 weeks following birth with a maximum peak at Week 1 (PRNT_50_ = 7156) and followed with relatively high titres until Week 5 (PRNT_50_ = 4608 [Figure 1D]).

After birth, foals are fully dependent on passive immunization from the progenitress’ colostrum, and this fact led us to carry out the microneutralization test with mares’ milk. Although, during the 6 weeks analysed, the neutralizing titre of colostrum was considerably lower than those found in the foals serum (maximum value of PRNT_50_ = 1408 and minimum = 128 [Figure 1E]).

**FIGURE 2:**
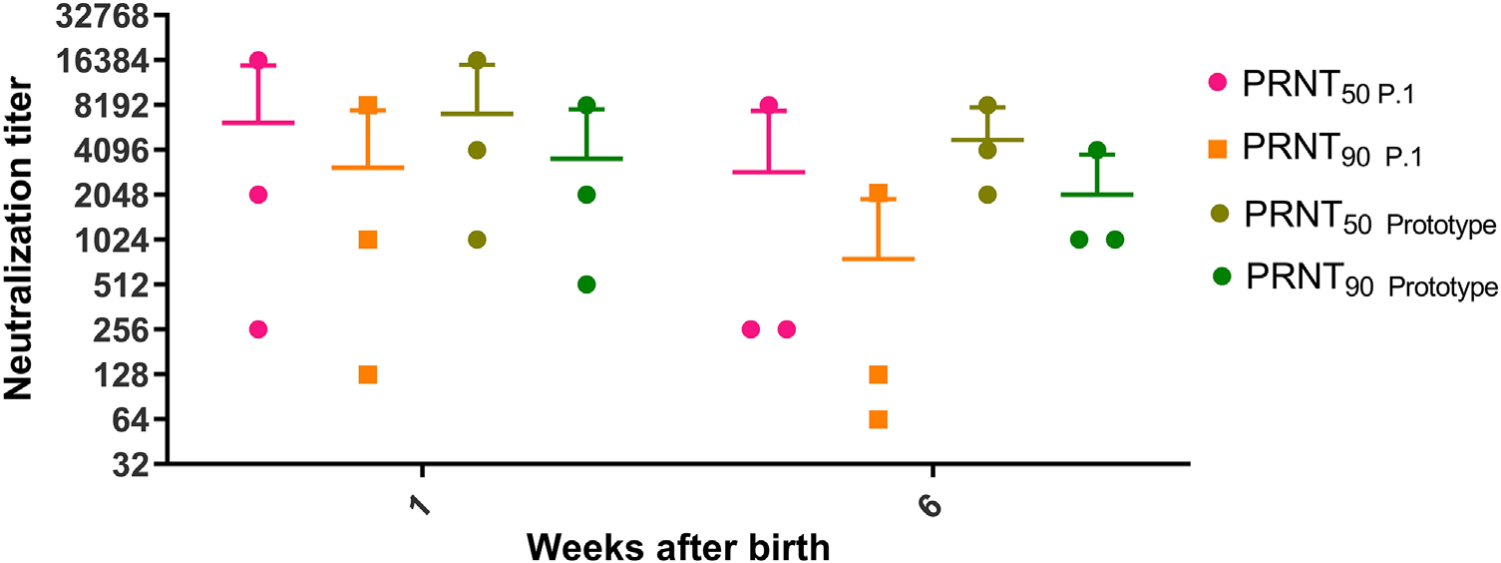
Microneutralization assays. PRNT_50_ and PRNT_90_ for foals serum collected on Weeks 1 and 4 after farrowing.

Finally, we analysed the neutralizing titre of mares’ serum and, as expected, the data showed very high neutralizing titres against the ancestral strain of SARS‐CoV‐2 during the entire period analysed, with PRNT_50_ = 17,408 for 1 week from birth of the foals. Even with a drop from the third week (PRNT_50_ = 6144), the high levels of neutralization remained at high levels from the antigen boost produced in Week 4 (PRNT_50_ = 10,204) with the maximum peak occurring 6 weeks after farrowing (PRNT_50_ = 18,432) (Figure 2C).

### 3.3 Immunoglobulins Transferred From Colostrum Also Neutralize the Gamma Variant (P.1)

Aiming to verify the neutralizing potential of immunoglobulins transferred by colostrum against SARS‐CoV‐2 variants of concern, we performed the foal serum microneutralization assay against the Gamma variant (P.1 in the pangolin classification), one of the variants with the highest number of mutated amino acids residues in the SARS‐CoV‐2 Spike protein structure (Garcia‐Beltran et al. 2021). The results showed that neutralizing titres remained high in the first week after birth (PRNT_50_ = 6229) and relatively high 4 weeks after birth in foals (PRNT_50_ = 2901) (Figure 2).

## 4 Discussion

One of the most interesting aspects found during the development of anti‐SARS‐CoV‐2 hyperimmune equine serum was the very high neutralizing titres that the sera from immunized animals reached (Cunha et al. 2021). In our previous report (Cunha et al., 2021), the data obtained by measuring the anti‐Sars‐CoV‐2 IgG titers of two different groups of animals showed specific antibody titers for the Sars‐CoV‐2 trimeric spike protein ‐ above 1:1,000,000 ‐ at the end of the first immunization cycle. Furthermore, the neutralizing antibody titers from the ten animals of the previous study after the first immunization cycle were equivalent to those found in the serum of the animals, both foals and mares, in the present report. High ELISA and neutralizing titers were described in similar studies using different antigens such as SARS‐CoV‐2 spike protein subunits (León et al., 2021; Zylberman et al., 2020) and inactivated whole viral particles (Botosso et al., 2022). Another report showed that passive immunization of horses using virus‐like particles (VLPs) containing MERS‐CoV structural proteins induced the production of high titers of both total and neutralizing antibodies (Zhao et al., 2017).

As soon as the pregnancy was verified, and after the birth of the foals as well, one of the curiosities that led us was the ability to transfer antibodies through colostrum intake. Due to the epitheliochorial diffusion in the mare's placenta, it is impossible for the large molecules to transpass the placenta and reach the foetus, and then, the passive immunization from the colostrum is critical to the development of the foal immunity (Jeffcott 1974a, 1974b; Perkins and Wagner 2015).

Our results corroborate to the literature with high specific SARS‐CoV‐2 spike protein immunoglobulins ELISA and neutralization titres found with regard to mare and foal serum during the weeks post parturition as shown previously (Baptista et al. 2020; Jeffcott 1974b; Perkins and Wagner 2015; Turini et al. 2020). In the literature, we have found several reports which point to critical immunoglobulin transfer in the first colostrum uptake by the offspring followed by a substantial IgG reduction 24 h after the parturition. Moreover, the post‐farrowing passive humoral immunization in foals is closely related to the macromolecular transfer through immature enterocytes in the distal small intestine (Weström et al. 2020). The literature indicates that the intestinal macromolecular transfer to foal occurs early after birth; thus, our data have shown very high neutralizing titres in foal serum during all the 6 weeks. This could be possible because, as described in the literature, those immunoglobulins transferred by colostrum have a half‐life about 23 days in the animal circulation, and the their levels keep decreasing constantly over the first 5 months of life (Baptista et al. 2020; Erhard et al. 2001; Jeffcott 1974b, 1974b).

Moreover, another important aspect that we wanted to assess was about sera neutralizing ability when challenged with the Gamma variant. The presence of high neutralizing titres in sera was not necessarily a surprise, considering that in previous studies by our group, we had already verified the neutralizing capacity of this variant in polyclonal antibodies from horses immunized with this same antigen (Cunha et al. 2021). Thus, these results represent a second proof of concept of the unequivocal source of the milk and offspring serum immunoglobulins.

On the other hand, our colostrum and milk IgG ELISA and microneutralization titres data were a little contrasting to the massive consensus in the literature. Our results have shown a considerable IgG titre not only on the offspring birth week, but also a persistent and significant SARS‐CoV‐2 IgG in the mare milk 6 weeks followed the parturition, whereas the literature indicates a significant reduction in total IgG concentration few hours parturition, as shown by the very first full article about equine passive immunization by Jefcott (1974a) and corroborate later by several reports (Baptista et al. 2020; Jeffcott 1974a, 1974b; Kohn et al. 1989; Pearson et al. 1984; Perkins and Wagner 2015; Turini et al. 2020). Here, it is important to highlight that the earlier studies have been used total IgG measurements in their inferences, whereas our study has measured an antigen‐specific IgG, what could explain the contrasting results among the studies—although was very unexpected for us the presence of high anti‐SARS‐CoV‐2 IgG in milk samples over the 6 weeks. Moreover, due to the strong total IgG decreasing over the first hours, all of these cited studies were only focused in the first hours or days of life, having a lack of a long‐term follow‐up of milk IgG levels. In addition, the microneutralization test of mares milk showed high titres of neutralization at first day and a significant decrease from the second week. However, this decrease was important, and it keeps very unexpected the persistent presence of considerable neutralizing titre—which is compared with the neutralization rate of samples from humans subjects vaccinated with similar antigen (but different adjuvants) (Ward et al. 2021).

Despite the compelling results presented here, some limitations prevent the broader interpretation of our findings. As this investigation originated from unexpected events during the routine activities on a farm dedicated to hyperimmune serum production, the study design lacked controlled experimental conditions. In order to validate and expand upon our findings, future studies incorporating well‐defined control groups, larger sample sizes, and standardized immunization protocols for pregnant mares are necessary. Nevertheless, our observations reveal the need for a better understanding of passive immunization in neonatal horses and, particularly in the context of managing donor animals for the production of hyperimmune serum. The evidence presented here suggests that immunization of pregnant mares may directly influence the antibody composition in the offspring’s serum. This phenomenon warrants careful consideration, as it may have significant implications for both the health of neonates and the efficacy of hyperimmune serum derived from such animals. A deeper understanding of these maternal‐fetal immunological interactions is therefore crucial to optimize practices in the field and prevent unintended consequences in the offspring.

## 5 Conclusions

In this study, we demonstrate the high prototype SARS‐CoV‐2 strain neutralizing capacity of immunoglobulins transferred through colostrum and milk from mares’ to offspring. One of the most important achievements of this study is to note the high presence of SARS‐CoV‐2 spike protein‐specific antibodies in the mare's milk and foal serum during the 6 weeks after parturition. In addition, the quantification of the IgG specific to an antigen previously inoculated only in the progenitors brings a little advantage to the large number of articles about the theme because it allows us to track specifically the antibodies that come from mares to foals, what could increase the knowledge about the passive immunization in equines. Thus, together, our data and methodology applied lead to propose this approach to better understand equine passive immunization.

## Author Contributions

**Victor Augusto Roncaglia‐Pereira**: conceptualization, data curation, formal analysis, investigation, methodology, validation, visualization, writing – original draft. **Carlos Henrique Dumard**: data curation, investigation, methodology, visualization. **Marcos Monteiro‐Machado**: data curation, visualization. **Paulo Melo**: data curation, visualization. **Juliana Fonseca**: data curation, visualization. **Leonardo Meirelles**: methodology, resources. **Luis Cunha‐Ribeiro**: conceptualization, funding acquisition, project administration. **Patrícia Souza**: data curation, visualization. **Jerson Lima da Silva**: conceptualization, funding acquisition, methodology, project administration, resources, supervision, writing – review and editing. **Leda Castilho**: methodology, resources, writing – review and editing. **Andréa Cheble de Oliveira**: conceptualization, data curation, investigation, methodology, project administration, writing – review and editing. **Andre Marco de Oliveira Gomes**: conceptualization, data curation, formal analysis, methodology, project administration, resources, supervision, writing – review and editing. **Marcelo Abrahão Strauch**: conceptualization, data curation, formal analysis, investigation, project administration, visualization, writing – original draft.

## Ethics Statement

All procedures involving animals were performed in accordance with the animal research ethical principles determined by National Brazilian Law 11.794/08 of the National Council for the Control of Animal Experimentation (CONCEA, Brazil). The protocol for the horses was approved by the Animal Care and Use Committee from IVB under permission no. 003.

## Conflicts of Interest

The authors declare no conflicts of interest.

## Peer Review

The peer review history for this article is available at https://publons.com/publon/10.1002/vms3.70488.

## Data Availability

Authors declare data will be available when requested.
